# Supplementary material for: Content-rich biological network constructed by mining PubMed abstracts
Source: BMC Bioinformatics. 2004 Oct 8;5:147. doi: 10.1186/1471-2105-5-147 (PMC528731; doi:10.1186/1471-2105-5-147)
Supplement: Additional File 2 — The original results of the above study (non-essential files are deleted to keep the file size under the limit set by BMC bioinformatics). [file 1471-2105-5-147-S2.bz2 › chilibotAdditionalFile2/dip05/587ID9546397E10956/html/HFE_B2M.html]

 


 **HFE** and **B2M** 
  
Found 31 abstracts in PubMed, retrieved 05.  
 

 What does Google say? 
 PDF only 
| .edu only 

---

**Interactive relationship** (e.g. stimulation, inhibition, etc)

**Neutral relationship**- **HFE**  is a nonclassical class I molecule that associates with beta 2 microglobulin  [ **B2M** ]  beta 2m and with the transferrin receptor.  Ref: 12456502 Blood, 2003
- Most patients are homozygous for a C282Y point mutation that abrogates the interaction of  **HFE**  with beta 2 microglobulin  [ **B2M** ]  beta 2 M and, thus, impairs its proper processing and expression on the cell surface.  Ref: 12464008 Biochem J, 2003

**Non-interactive relationship** (e.g. studied together, co-existance, homology, etc.)

- To determine its role in hereditary hemochromatosis  [ **HFE** ]  HH, we used beta2 microglobulin knockout  **B2M**  mice that accumulate iron as in HH.  Ref: 12181184 Am J Physiol Gastrointest Liver Physiol, 2002
- The haemochromatosis  [ **HFE** ]  protein  **HFE**  induces an apparent iron deficient phenotype in H1299 cells that is not corrected by co expression of beta 2 microglobulin  [ **B2M** ] .  Ref: 12464008 Biochem J, 2003
- The following candidate genes have been examined in detail to determine whether polymorphisms in them may be responsible for this variation transferrin, transferrin receptor 1, transferrin receptor 2, ferritin L, ferritin H, IRP1, IRP2,  **HFE** , beta 2 microglobulin  [ **B2M** ] , mobilferrin calreticulin, ceruloplasmin, ferroportin, NRAMP1, NRAMP2 DMT1, haptoglobin, heme oxygenase 1, heme oxygenase 2, hepcidin, USF2, ZIRTL, duodenal cytochrome b ferric reductase DCYTB, TNFalpha, keratin 8, and keratin 18.  Ref: 12547238 Blood Cells Mol Dis< MedlineTA>Blood Cells Mol Dis, 2003
